# Thanks for hearing me: key elements of primary care according to older patients

**DOI:** 10.1080/02813432.2024.2317833

**Published:** 2024-02-21

**Authors:** Lisa Kastbom, Maria M. Johansson, Annette Sverker, Anna Segernäs

**Affiliations:** a Department of Health, Medicine and Caring Sciences, Linköping University, Linköping, Sweden; b Primary Health Care Centre in Ekholmen and Department of Health, Medicine and Caring Sciences, Linköping University, Linköping, Sweden; c Department of Activity and Health in Linköping, and Department of Health, Medicine and Caring Sciences, Linköping University, Linköping, Sweden; d Department of Acute Internal Medicine and Geriatrics, and Department of Health, Medicine and Caring Sciences, Linköping University, Linköping, Sweden; e Pain and Rehabilitation Center, and Department of Activity and Health, and Department of Health, Medicine and Caring Sciences, Linköping University, Linköping, Sweden

**Keywords:** Older adults, patient-centered care, patient participation, primary health care, qualitative research

## Abstract

**Objective**: When organising healthcare and planning for research to improve healthcare, it is important to include the patients’ own perceptions. Therefore, the aim was to explore older patients’ views on what is important concerning their current care and possible future interventions in a primary care setting.**Design**: A qualitative design with individual interviews was used. Analysis through latent content analysis.**Setting**: Seven Swedish primary care centres.**Subjects**: Patients (n 30) aged >75 years, connected to elder care teams in primary healthcare.**Results**: Three categories, consisting of 14 sub-categories in total, were found, namely: Care characterised by easy access, continuity and engaged staff builds security; Everyday life and Plans in late life. The overarching latent theme Person-centred care with easy access, continuity and engaged staff gave a deeper meaning to the content of the categories and sub-categories.**Conclusion**: It is important to organise primary care for older people through conditions which meet up with their specific needs. Our study highlights the importance of elder care teams facilitating the contact with healthcare, ensuring continuity and creating conditions for a person-centred care. There were variations regarding preferences about training and different views on conversations about end-of-life, which strengthens the need for individualisation and personal knowledge. This study also exemplifies qualitative individual interviews as an approach to reach older people to be part of a study design and give input to an upcoming research intervention, as the interviews contribute with important information of value in the planning of the Swedish intervention trial *Secure and Focused Primary Care for Older pEople* (SAFE).

## KEY POINTS

- In this qualitative interview study, the three categories *Care characterised by easy access, continuity and engaged staff builds security; Everyday life* and *Plans in late life* with underlying sub-categories describe the older patients’ views on what is important in their current care and possible future interventions in a Swedish primary care setting.
- The latent theme *‘Person-centred care with easy access, continuity and engaged staff’* was formed to give a deeper meaning to the content of the categories and sub-categories.
- Having a permanent care contact with a responsible nurse in an elder care team and a personal doctor (most often a general practitioner) over time seems important for vulnerable older patients with high risk of hospitalisation.
- The study suggests qualitative individual interviews as an approach to increase older peoples’ participation in future clinical complex study designs.

## Background

Previous research has shown that patient care improves when health care interacts with patients [1]. Patient participation is a general term describing participation as an active part in one’s own care for the patient and his or her family members. Establishing patient partnership in primary care also offers opportunities for high-quality patient-centred care [2]. Different examples of patient and public involvement (PPI) in research have been described, such as panels for research priorities, and support in the study design of research methods, or in advisory groups, often with improved research as reported outcomes [3]. There are several reasons for patient involvement in research, namely: moral reasons, methodological reasons and policy reasons and several models have been described previously, such as focus-group interviews, fully embedded or reaching out for specific groups of interest for the study or hard-to-reach groups [4]. Vulnerable patient groups, such as older people with frailty and cognitive decline, or mental health issues, are considered particularly challenging in active patient involvement due to their special needs, which is why they are often excluded [5,6].

Due to the complexity of older people, including multimorbidity, polypharmacy and cognitive impairment, the holistic organisational form of care called ‘Comprehensive Geriatric Assessment’ (CGA), is highlighted as important and regarded as the gold standard in hospital geriatric care [7,8]. However, the model is less studied, and with diverse results, in outpatient care, especially primary care [9]. Because of the difficulties in identifying individuals who may benefit from CGA, there have been attempts to use administrative data for the identification of high-risk patients [10]. In the previous Swedish study *Proactive healthcare for frail elderly persons*, patients aged from 75 years in one region were selected using a prediction model, which estimates the risk of hospitalisation from electronic medical records [11–14]. The intervention involved a specialised *elder care team* (in Swedish: *äldrevårdsmottagning*) at each primary care centre, consisting of a nurse and a physician who made a CGA of each patient by using the Primary Care Assessment Tool for the Elderly (PASTEL), created to include physical, social and psychological items [12,13]. There was no standard treatment after the assessment. Positive results with reduced hospitalisation in the intervention group and preliminary cost-effectiveness were shown [15]. However, knowledge is scarce regarding the individual specific care interventions implemented for the older patients in the primary care CGA model, as well as what actually benefits the patients from their own point of view.

Previous research has pointed out that except for medical treatment, including having the optimal medication [16], both mental and physical health, including social contacts, everyday life activities and training, is of importance for older persons to remain health and quality of life in later life [17–19]. Further on, participation in care and planning for end-of-life (EOL) is recommended [20]. To enable this, teamwork is necessary.

The intervention trial *Secure and Focused Primary Care for Older pEople* (SAFE) is a follow-up and expansion of the previous study *Proactive healthcare for frail elderly persons*, with the ambition to evaluate a customised and person-centred primary care CGA working model, possible to implement broadly in general practice in close collaboration with surrounding healthcare actors. The objective is to obtain additional data on large-scale implementation of the model and to evaluate the cost-effectiveness in a larger primary care population in several Swedish regions, as well as to study the experience of increased participation of patients and their family members in care and care planning. The intervention includes different components, and the study design can be seen as an example of a complex intervention. According to the new framework for developing and evaluating complex interventions, a core element is to engage the stakeholders in the development of the study intervention [21]. This new framework by Skivington et al., emphasising the importance of engaging stakeholders (i.e. the vulnerable older persons in primary care) in the planning of a complex study design, together with an evidence based geriatric holistic view, constitutes the epistemological approach in the present study.

Out of patients’ experiences from *Proactive healthcare for frail elderly persons*, there was the possibility of increasing patients’ involvement in the research planning of SAFE. The patient participation is primarily aimed at leveraging the patients’ experiences of the elder care teams, and to further explore which care efforts older patients want to maintain and encourage in the future planning of the SAFE intervention. Experience from previous PPI studies among older, vulnerable patients highlights that PPI methods need to be flexible and appropriate for the research context [6].

Framed in this context, qualitative individual interviews in the SAFE study planning give the opportunity to gain knowledge of the older peoples’ perspectives on interventions and aspects of outpatient care, beyond the researchers’ perspective, and to improve the study protocol and the development of the intervention in the SAFE trial. Furthermore, patients will have the opportunity to contribute with their experiences of elder care teams in primary care, and share their views regarding their diseases, treatments, daily activities, proactive care planning and EOL questions. Therefore, the aim of the present study was to explore older patients’ views on what is important as regards their current care and possible future interventions in a primary care setting.

## Material and methods

### Participants and interviews

Inclusion criteria were that the participant should be a patient at any of the nine primary care centres that had participated in the intervention *Proactive healthcare for frail elderly persons* [11–13], be Swedish-speaking and accept that the interview would be recorded. Participants were recruited through a data search of the Care Data Warehouse (in Swedish: *Vårddatalagret*), which contains all contacts with healthcare in Region Östergötland (the county council). The data search selected 50 patients from seven primary care centres that had taken part in the intervention, all of whom were invited to participate. The responsible nurses at the primary care centres assessed whether the selected patients were able to participate in a telephone interview. Only those who were assessed by the nurses to be capable of taking part were asked to participate in the study. To ensure a wide variety of participating patients, both urban (n 5) and rural (n 2) primary care centres were represented from all three areas of the region [22]. Of the 50 individuals who were asked to participate in the study, 30 agreed to take part. Frequently mentioned reasons for refraining from participating were poor health and having participated in previous studies. The mean age of the participants was 86 years (range 75 – 96). Eighteen (60%) were women, and twelve (40%) were men.

An interview guide (which is available on request) was constructed by the research group. This consisted of open questions concerning each patient’s everyday life and function, views on current care and possible future interventions at the elder care teams, and how they related to the future and EOL issues, such as proactive planning. To minimise the risk of ambiguity in any of the statements made by the participant, clarifying questions were asked [22]. The guide was tested on three pilot interviews performed by all the three interviewers, and subsequently thoroughly discussed by the entire research group who reached consensus that the interview guide was considered comprehensive with understandable questions. The pilot interviews were included in the study since the data were considered rich.

The interviews were performed between November 2021 and January 2022. Three of the researchers (AS, MJ and LK), all with experience in qualitative research but from different scientific disciplines, such as health, ageing, and social work research, conducted the interviews (AS: n 11, MJ: n 9, and LK: n 10). The interviewers consisted of one general practitioner (GP), one social worker and one occupational therapist. In total, 30 participants were interviewed individually. The different experiences and backgrounds strengthened the reflexivity process [22]. Common characteristics of the participants were old age, multimorbidity and frailty to varying degrees. Commonly mentioned conditions and diseases were cardiovascular diseases, such as hypertension, heart failure, coronary disease, diabetes mellitus and stroke. Pulmonary diseases, such as chronic obstructive pulmonary disease and kidney failure were also common, according to the participants. Functional impairments, such as fear of falling, pain, reduced muscle strength and energy loss, impaired balance and dizziness, worries, hearing and vision impairment and changed sleeping patterns were also frequently described. Due to the COVID-19 pandemic, the interviews were carried out by telephone. None of the informants dropped out at any phase of the study. Participant inclusion continued until redundancy appeared [22], i.e. when discussing the content of the conducted interviews, the four researchers agreed that additional interviews would add no new perspectives on the questions related to the aim of the study. The interviews were digitally recorded and transcribed verbatim by professional transcribers employed by an external company.

### Analysis

The interviews were analysed through qualitative content analysis with no predetermined categories or themes [23]. The analysis went through the following seven steps: (1) The transcribed interview was read through to obtain an overall impression and to get a broad understanding. (2) Segments of the texts dealing with the aim of the study were identified and meaning units were constructed. (3) The meaning units were condensed and abstracted to codes. (4) The codes were compared and sorted to sub-categories and categories. (5) The categories were compared to the entire interview, to make sure that the interpretation was consistent and coherent with the text as a whole. The categories were compared to avoid overlapping and content descriptions were developed. (6) One latent theme was formed to give a deeper meaning of all the categories in the analysis. (7) Quotations were used to exemplify the results [23]. Examples of the steps of the analysis are shown in Table 1.

**Table 1.** Steps of the qualitative analysis.

Meaning unit	Code	Sub-category	Category
… ‘and it felt very safe, and it was completely different from being in the queue system and needing to explain one’s matter before getting to the right person … I think it’s a fine system’.	Safe. No queue system. Fine system.	Easy access rather than telephone queue system	Care characterised by easy access, continuity and engaged staff builds security
‘I do all the cooking and it works well, but it is …. I feel that the energy increasingly disappears, so it’s probably a matter of time to have the energy to do it all. And all the papers and such things coming. I have got a daughter and they live north of Stockholm, so I haven’t got that much help ‘.	Doing all the cooking works well. Energy disappears. Daughter far away. Not much help.	Everyday activities and assistive devices	Everyday life
… ‘I only hope that it goes quickly. I’ve said that to my family here … I wouldn’t like to be a vegetable. If that’s the case, I’d prefer a quick end and so. That’s my thought’.	Thoughts on dying. Hoping for a quick end. EOL wishes. Don’t want to be a vegetable. Share one’s thoughts with family.	On death and dying	Plans in late life

The preliminary categories were coded by the researchers who performed the interviews, i.e. the three researchers performing the interviews read all the 30 interviews. A few of the interviews were coded by all three interviews separately. Preliminary coding of all the data was made by LK. The preliminary categories were further discussed and revised by all the researchers. As part of the reflexivity process, the categories were validated by supplementing and contesting each other’s readings and preunderstandings [24]. The results of the coding and different views and meanings were discussed by the whole research group until agreement was reached, as part of validating the findings [22].

## Results

**Figure 1.**
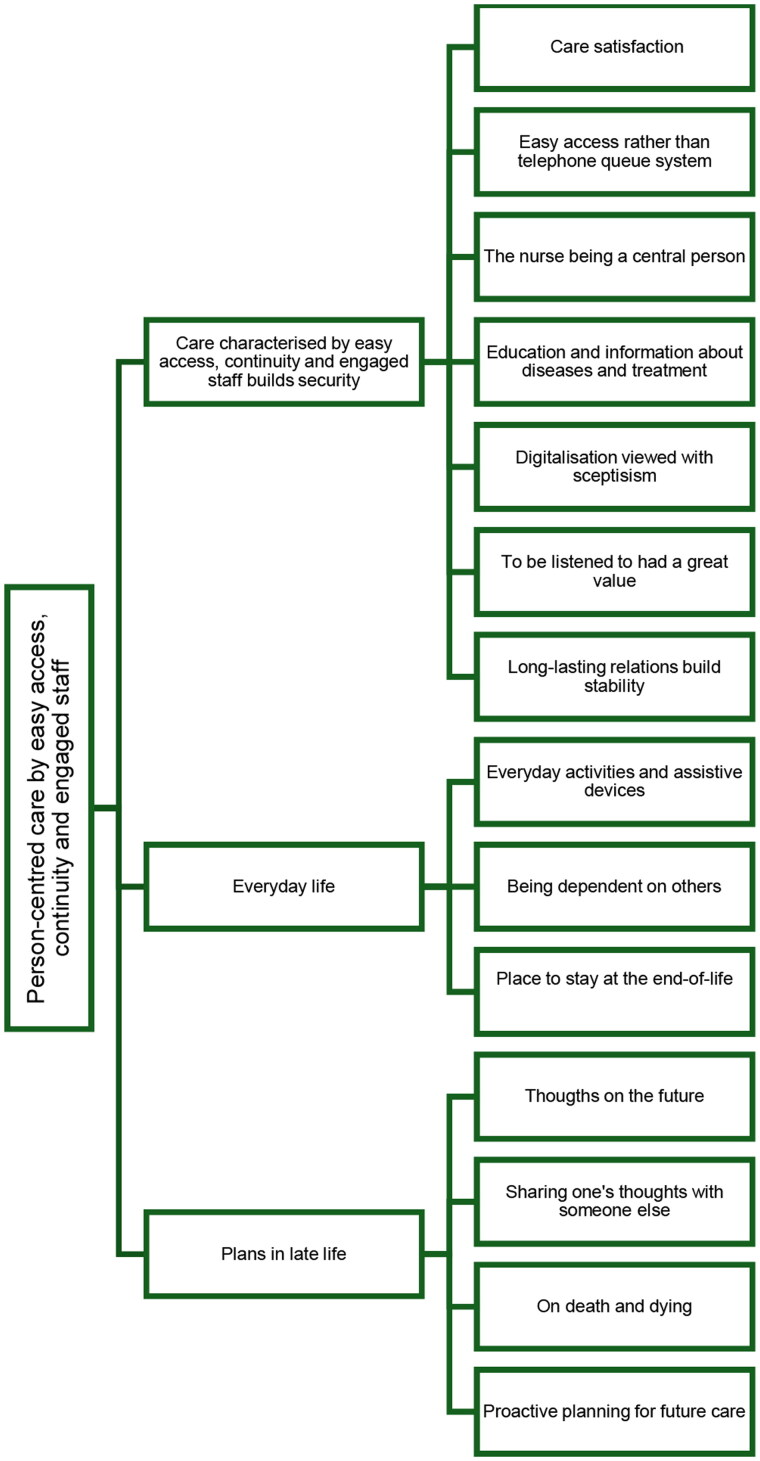
Overview of older patients’ views on what is important concerning their current care and possible future interventions in a primary care setting. All three categories and 14 sub-categories are related to the latent theme *Person-centred care with easy access, continuity and engaged staff.*

When analysing the data through qualitative content analysis [23], three categories describing older patients’ views on what is important concerning their current care and possible future interventions in a primary care setting were seen, namely: *Care characterised by easy access, continuity and engaged staff builds security; Everyday life* and *Plans in late life*. These categories consisted of 14 sub-categories in total. One overarching latent theme was formed to give a deeper meaning to the content of the categories, namely: *Person-centred care with easy access, continuity and engaged staff* (see Figure 1). The quotations are presented with the initials of the interviewers (AS, MJ or LK), followed by participants’ numbers.

### Care characterised by easy access, continuity and engaged staff builds security

#### Care satisfaction

Participants expressed satisfaction and appreciated the care received from their primary care centres in general, and the elder care teams in particular. They described a positive progress of the care by such teams. As the participants were consistently satisfied with the care received from the elder care teams, they had difficulties in formulating any changes for improving and developing the concept.

[On the elder care team] And yes, I think I perceived it as very positive. I think it’s the best thing they have done for the older ones. (LK 07. 88-year-old woman.)

#### Easy access rather than telephone queue system

Being able to contact the elder care team directly, through an easy access telephone number to the team nurse, rather than using the regular telephone queue system, was associated with feelings of convenience, safety, and security. This was mentioned by almost every participant.

… and it felt very safe, and it was completely different from being in the queue system and needing to explain one’s matter before getting to the right person … I think it’s a fine system. (AS 25. 95-year-old man.)

#### The nurse being a central person

The nurse was described as an important and coordinating person with competence and knowledge of the older peoples’ needs and was further viewed as someone who understood the importance of offering the patient safety, security and empathy with the patient being central. The nurse was seen as someone who could solve any issues and pass questions on to the physician, or other professionals, when she/he needed further assistance.

Yes, there is a special phone number which I dial and most often I might get a response immediately or they call me back later on and so on, but it works really well and if there is something that needs a physician’s input, that will happen and thus I get a response. (LK 27. 85-year-old woman.)

#### Education and information about diseases and treatment

A review of the participants’ diseases and ongoing treatments was welcomed, to better understand the course and expected progress of the diseases, and the action of medicines.

[on a review of the patient’s treatment by the physician] Yes, that would have been great! Absolutely. But there is … there is always this feeling … that there is no time to do this. I have always got this feeling that they have so much work to do. There’s not lots of time. (LK 23. 95-year-old woman.)

Some expressed that they received the information they needed on these matters from their physician regularly. Others lacked such information or found that they had insufficient knowledge on their own medication and the purpose of different treatments, thus expressing a wish for education and clarification on diseases and treatment to be a future component of their care.

Well, I can say I haven’t got any information on that. I haven’t. I have had some severe diseases that I have had surgery for. But I haven’t got any information about it … I can’t say that I have. (LK 04. 86-year-old man.)

#### Digitalisation viewed with sceptisism

Digital means of contacts and making appointments were dismissed by most of the participants, since they preferred talking to a person, a physical contact, or because they were unaware of digital methods. However, some expressed curiosity when talking about for example using digital methods for coaching in physical activity.

I don’t know. I haven’t thought about that. How does it work then? By computer for example?//Yes, actually it could be good really. (AS 21. 88-year-old woman.)

#### To be listened to had a great value

The participants did not want to contact the primary care centre unnecessarily and avoided burdening healthcare. They appreciated the care of older people by committed and interested healthcare staff, characteristics primarily described when talking about the team nurses. Being given enough time and being listened to could alleviate negative feelings of being a burden, according to the participants.

Yes, when you’re this old, you don’t make a doctor’s appointment or ask for a visit if you aren’t severely ill. And then being made this welcome and being invited to … yes, sit down and talk [to the elder care team nurse]. That is absolutely fantastic, I think. That, I really appreciate. (LK 07. 88-year-old woman.)

#### Long-lasting relations build stability

Continuity was highly valued and patient-physician relationships that had continued for years meant that the participants felt safe and secure.

Then I see her [the physician] regularly every six months, unless there is something coming up that I need to … I can contact a nurse who passes the message to the physician, and it works really well. (AS 20. 85-year-old woman.)

### Everyday life

This main category consisted of three sub-categories describing the content of the participants’ days, as well as their everyday activities, function, needs of assistance from others, and thoughts on a place to live.

#### Everyday activities and assistive devices

Variations regarding function and activity in everyday life among the participants were expressed. Some still managed to take care of themselves, which included cleaning, cooking, and paying bills, while others needed assistance through municipal home care or support from their family members.

I do all the cooking and it works well, but it is …. I feel that the energy increasingly disappears, so it’s probably a matter of time to have the energy to do it all. And all the papers and such things coming. I have got a daughter and they live north of Stockholm, so I haven’t got that much help. (LK 09. 94-year-old woman.)

Ability and motivation to be physically active also varied, from being inactive and immobile to exercising on a daily basis. Some of the participants had former or current contact with physiotherapists and/or occupational therapists, while others had not been offered such interventions, although they expressed a desire for these when being interviewed. Reduced energy and a fear of falling were obstacles for exercising on their own. Doing their everyday life activities was also often seen as an exercise itself, and a wish for retaining function and fighting for independence as long as possible was expressed.

Assistive devices, such as rollators, freestanding shower chairs, and home adaptations could facilitate everyday life.

I have got a rollator with a tray, but I don’t use it that much. Only in periods when I think I am shaky, that I can use it in the bedroom and use it when I walk to the bathroom. But I don’t use it right now. (AS 11. 87-year-old woman.)

#### Being dependent on others

Assistance and support from others were associated with gratitude, but it could also cause thoughts of being a burden to family members and other people of importance to participants, who did not want to be dependent on others.

… Before, I used to be able to manage on my own, and now suddenly I can’t. Then I have to ask for help.// [on being dependent on others] Well, it’s not a good feeling. One tries to manage as well as one can. But it won’t help now. Because it doesn’t work. (LK 01. 81-year-old man.)

#### Place to stay at the end-of-life

Some had positive experiences from others’ stays in nursing homes at the end of their lives and could accept moving into such institutions when their health began to deteriorate.

Oh yes. I have been to nursing homes. My mother-in-law and my mum stayed there, and it was clean, and tidy, and nice, I think. They’ve got their rooms and their … yes. They bring some things from their homes, so I think it looked really nice. When you are this old, you don’t make demands about anything, really. (LK 04. 86-year-old man.)

However, for others, living in their ordinary homes until the end was essential and these participants wished to stay at home until their death. For some, there was uncertainty concerning how to manage to stay at home while deteriorating, and for them, moving into a nursing home was seen as a necessary evil at the end of their lives.

By then, it will be difficult to be at home. Then, I have to move into a nursing home, I guess. It’s nothing that I want. (LK 01. 81-year-old man.)

### Plans in late life

Since the participants were all old, there was an awareness that the end of their lives was approaching. There was a variation regarding participants’ views on, and planning for, their remaining lifetime.

#### Thoughts on the future

Some of the participants tried to put thoughts of the future aside, since such thoughts could cause anxiety and sadness. Deteriorating health and increased fatigue sometimes triggered feelings of meaninglessness.

I try to take one day at a time and not grieve too much. Sometimes, the thoughts come to me. I try to put them out of my mind … But it is the fact that one is getting worse and worse. Coping with less. It feels so meaningless sometimes. (LK 01. 81-year-old man.)

Others were looking forward to events ahead, such as social gatherings, indicating that plans for the future still existed and were important, despite their knowledge of having a limited remaining lifetime.

This summer, if it works out, we are having a great garden party in the garden, and I will invite all the children and grandchildren and great-grandchildren, and there are going to be more than thirty of us! (AS 14. 89-year-old woman.)

For some, thinking about the EOL was peaceful, and death was described as a natural part of life, not necessarily associated with distress.

You are finished by then, yes. You just have to look ahead and gradually wait for it to come. But I don’t think that is strange. For me, it’s quite natural. (AS 22. 77-year-old woman.)

Some participants welcomed death, sometimes having the belief that after death there is something else, maybe reunions with loved ones who had already passed away.

Well, firstly it means that I have not lost hope of seeing my wife again … and it also means that I think that I don’t think that everything totally finishes after death … but somehow it all carries on … (AS 25. 95-year-old man.)

Preparing for death through facilitating for families by practical tasks or by sorting out financial affairs was important for some participants.

My son, who I hope will take over, he is not that practical, so I must sort things out as good that I can …. And there is some painting that I can partly do on my own and partly will need some help with. But I want to have it done anyway … (AS 31. 89-year-old woman.)

#### Sharing one’s thoughts with someone else

Sharing wishes and thoughts about EOL with family members occurred, as well as the opposite. Sometimes, family members had asked for conversations and plans about the future, although the participants wished to postpone such talks, in some cases to prevent their own discomfort.

My daughter has gone on about it, about sitting down to write something, but I don’t think I’m there yet//to write how I want it to be … But … But we can do it later. (LK 02. 86-year-old man.)

Another reason to suspend EOL conversations, was to protect family members, since these issues could cause sadness, according to some of the participants.

I don’t think many reflect on this before. One puts it off, postpones it.//Yes, not yet … not yet … not yet. Yes, right. But it’s probably the same … I think it’s the same for the children. They don’t want to touch on it either.//Yes. But you must try, I guess. (LK 23. 95-year-old woman.)

#### On death and dying

Although some did not want to think about EOL issues, even less have such conversations with others, some of the participants had specific wishes concerning the time preceding death and the moment of death and had shared their thoughts with their families. One participant expressed worries of dying alone.

The only thing that I’m scared of is to lay at home and die and that they won’t find me for a few days. That’s what I think of most in that case. I have some daily visits from friends and so on. They call me every day och if I don’t answer, they usually get worried, actually. So, I have got some eyes on me. But about end-of-life … I would just like to have someone holding my hand. Somewhere, yes … (MJ 05. 86-year-old woman.)

Someone else hoped it would all go quickly, rather than a drawn-out process at the end.

… I only hope that it goes quickly. I’ve said that to my family here … I wouldn’t like to be a vegetable. If that’s the case, I’d prefer a quick end and so. That’s my thought. (AS 15. 90-year-old man.)

#### Proactive planning for future care

According to some, conversations about EOL and proactive planning with healthcare staff were associated with unease, as questions about EOL were felt to be private. For these participants, EOL questions were not dealt with other than with family members, or maybe not with anyone at all.

Completely unnecessary, I reckon [on healthcare staff from primary care raising EOL questions]. It also touches on one’s personal life too much. (MJ 30. 82-year-old woman.)

On the other hand, some had discussed such matters with their physician and knew that their preferences were documented in their electronic health records.

Yes, a physician that I’ve got, he knows it.//Now, it’s been a while since we talked, but it is in my health records. (MJ 17. 96-year-old woman.)

A few specifically asked for their autonomy to be respected right until the end by sharing their wishes and by participating in the planning of their care ahead.

Well, I have said how I want it to be, so I hope this will be respected. (AS 22. 77-year-old woman.)

## Person-centred care with easy access, continuity and engaged staff

The overarching latent theme *Person-centred care with easy access, continuity and engaged staff* was formed to give a deeper meaning to all the three categories describing older patients’ perceptions of what is important concerning their current care and possible future interventions in a primary care setting (see Figure 1).

The participants described that the group of older individuals that they represented needs clarity, together with support that helps them contact their primary care centres when needed. Although the older persons were sometimes rather hesitant to contact healthcare as they did not want to be a burden and use care unnecessarily, they appreciated being treated with respect, being given sufficient time, and being listened to attentively in conversations and appointments in primary care. This was offered by the elder care teams.

We are also so honoured to have an elder team nurse who is so very good and treats older people … I turn 100 soon … with respect and empathy … (AS 31. 89-year-old woman.)

Views concerning some of the aspects of the organisation of primary care, such as easy ways to get in touch with healthcare staff through a direct telephone number rather than a queue system and the appreciation of a having a permanent care contact with a responsible nurse in an elder care team were general and recurrent. Their views on other aspects, such as preferences regarding need and amount of information about diseases and treatment, and on digitalisation varied more in relation to person-centred care.

The participants felt that, to a great extent, the elder care teams provided the person-centred care suited to the needs of older people, which they had expressed a desire for. However, there were variations regarding wishes and preferences about both training and assistance in daily life. For some, assistance and support were required to be able to stay in their ordinary homes and for experiences that gave quality of life to older people. For others, being dependent on somebody else caused feelings of discomfort and guilt.

Well, I can’t ring my neighbour and tell him or her to take care of me and help me. I can’t ask for that, for others to take care of me. I must manage by myself. (LK 26. 87-year-old woman.)

Similarly, views on conversations about EOL and death with family members and healthcare staff and wishes to be involved in the planning of their future care also varied individually among the participants. Some had shared their wishes with both family and staff, and knew that their preferences were documented in their electronic health records, while others trusted family members, and assumed that others knew their preferences although they had never had such conversations. Sometimes, they did not want to think about EOL or share their thoughts, and some postponed such conversations till later. Other participants felt that avoiding letting the family members know one’s wishes regarding EOL and death was irresponsible.

Yes, well, that one must do, you know. Many, they don’t do it, yeah. And then family members are standing around like question marks asking how he wants it to be. No, it has to be sorted out so that they know, and I have told the family that I want to get cremated, you know. (LK 06. 76-year-old man.)

## Discussion

### Statement of principal findings

This study highlights that for older people, person-centred care and individualisation seems particularly important in meeting each patient’s specific needs. Older patients need clarity and support to facilitate the contacts with healthcare. The results also highlight the need for continuity of care and personal knowledge of the patient. Older patients express that elder care teams, through good personal knowledge of the patients within the team and personal continuity in the professional care relationship, could achieve this, with a feeling of safety and satisfaction within primary care. Having a permanent care contact with a responsible nurse in the elder care team and a personal doctor (most often a general practitioner) over time seems important. This has also been seen in previous studies [25,26]. There is variation regarding older people’s needs of assistance and support in everyday life and concerning their wishes to share EOL thoughts and preferences for future care. Physical activity should be seen in the patient’s everyday context and training advice needs to be personalised. Accessibility of professional support is an important aspect as well. This is in line with previous results from the *Proactive healthcare for frail elderly persons* study [27]. It seems important to organise care for older people in a manner that facilitates contacts with care units and also by creating conditions to meet their specific needs. This has also been pointed out in previous studies [25,28]. Personal telephone contact instead of general telephone advice is clearly preferred. The interest shown in digitalisation is minor within this patient group. Information and communication technology (ICT) is also known to be less used by frail older people [29]. Healthcare staff involved in the care of older people should consider that needs, thoughts, values, and preferences expressed by one patient might not be shared by others [30]. Each patient should be asked, sometimes repeatedly, about specific needs and wishes and what is important for them in the end of their lives. This requires both continuity and humility in raising sensitive questions repeatedly, as the patient’s views and wishes may change over time [31–33].

This study exemplifies qualitative individual interviews as an approach to reach out to old patients with some degree of frailty, to be part of a study design and to give their input to an upcoming research intervention. The results provided an opportunity for old patients at high risk of hospitalisation and mortality, to express their views on the planned intervention and future care in the SAFE trial. This might be one possible way of increasing patient participation in complex study designs, aiming to reach specific vulnerable patient groups. We perceive that the qualitative interviews in the planning and preparation of the SAFE trial have contributed with important information, from the patients’ perspective, of value since participation of patients and relatives in care and care-planning, as well as person-centred care, are core elements in the SAFE study design.

### Strengths and weaknesses of the study

One strength of the present study was the involvement of several scientific disciplines (i.e. one social worker, one occupational therapist and two physicians who were general practitioners) in the research group during all stages. The interviews were performed by a researcher without personal knowledge of the participant and, if clinically active, working at a different primary care centre than the participant. By involving researchers with different scientific backgrounds and professions, the approach to interviewing probably resulted in a broad range of subject matter being highlighted, thus giving a greater depth to the data. A GP with experience in the care of older patients in primary care, one social worker and one occupational therapist, also with clinical experience of older patients, performed the interviews. This could have influenced the answers of the participants. However, all authors were aware of this risk, and the interviewers tried to stay neutral to it during the interviews as well as in the process of the analysis, thus being a neutral investigator [22].

The participants had an active relationship with their elder care teams during the interview period. This gave the participants the possibility to reflect on the elder care teams. Also, since the elder care teams had been up and running for a few years, they had a good comprehension about the form of care from these teams in comparison to the regular care provided by the primary care centre. On the other hand, the fact that they were connected to the elder care teams and were satisfied with the care received by these teams might have led to difficulties in formulating possible ways to improve the care and present ideas for future interventions in primary care, which could be seen as a limitation of the study. Of the 50 individuals who were asked to participate in the study, 30 agreed to take part. One reason for refraining from participating was poor health. This could be seen as a limitation of the study since it could have affected the results. However, according to the authors, including participants with too little energy to manage an interview situation, would probably cause these individuals more harm than benefit.

Participants were recruited from both urban and rurally located primary care centres in all three areas of the county studied. This gave a broad variety of participants, supporting the generalisability of the findings. Another strength is the large number of interviews with rich data to analyse.

All the interviews were performed over the telephone. This may have resulted in more honest answers, as the participants did not have to be face-to-face with the interviewer, thus minimising the discomfort when talking about sensitive issues [34,35]. However, it may also have led to less dynamic conversations, as the non-verbal aspect of the conversation was missing. Sensitive topics, such as EOL conversations over the telephone, could lead to unease. However, the interviewers kept this in mind and were all perceptive and sensitive to any possible reaction of the participant. The three researchers who performed the interviews were all experienced interviewers, with previous experiences of both interviews with older people and interviews focusing on EOL issues. As a result, during each interview, there was an increased sensitivity to the participant not wanting to talk further about the question or subject.

### Findings in relation to other studies

Improving healthcare safety, quality, and coordination, as well as quality of life, are important aims of caring for older patients in primary care. Person-centred care is one approach to reach these aims [36]. However, there have been difficulties in finding standardised, agreed-upon parameters for such care. Person-centred care supports individuals’ choice and autonomy in healthcare decisions, and it has become an important avenue for improving primary care [37]. One definition means that individuals’ values and preferences are elicited and once expressed, guide all aspects of their healthcare, supporting their realistic health and life goals. This requires dynamic relationships with caregivers, as well as involvement and collaboration with other people of importance to the old patient, which is also shown in the present study. Other studies focus on the value of shared decision making in care planning as part of person-centred care and interdisciplinary teamwork [38]. We propose elder care teams in primary care, with continuity in care contacts with responsible nurses in the elder care teams and a personal doctor, to facilitate care in line with the key elements of person-centred care; namely communication, coordination and team-based care [39]. The importance of continuity in primary care has previously been shown in Sweden [40].

### Meaning of the study: possible mechanisms and implications for clinicians or policy makers

To fulfil the needs and wishes of old patients, primary care must be organised in a person-centred manner and with a focus on continuity. Close collaboration and coordination with community care and rehabilitation is necessary since both care and physical activity and training need to be individualised. Short paths between assessment, intervention, and reassessment in the event of a change in condition are desirable. An understanding of the differences in function and activity seen within the group of old patients is necessary to manage personalisation of care and support. There are possible barriers to successful implementation in primary care, such as organisational cultures, physicians’ workloads, inadequate indicators for healthcare measurements and financing, as well as ethical concerns when a person makes a decision that the healthcare provider strongly disagrees with, and that puts the person at significant risk [37]. There are also facilitating factors, through stakeholders’ person-centred knowledge, respecting the autonomy of older people, and strengthening multidisciplinary team members, for implementation among community-dwelling old people [41]. Staff training to increase knowledge and support working in teams with this patient group should be prioritised.

Respectful treatment and gentle listening in a long-lasting care relationship based on continuity and flexibility, are important components of the care of old patients. These patients express wishes to receive information about the content and direction of care and often prefer to participate in their care, but to varying degrees. Overall, person-centred care and activities adapted to everyone’s needs and wishes, with an awareness that the patient’s views could change over time, emerged. The old patients describe primary care adapted comprehensive geriatric assessment (CGA) with PASTEL perceived as positive and underlined the importance of caregivers with specific knowledge about ageing. Elder care teams at the primary care centres could, together with a physician, support continuity over time.

It seems challenging to facilitate a holistic person-centred care with the opportunity for the patient to be listened to, whilst having a resource-efficient approach in primary care. Primary care population intervention studies, which include implementation aspects, are of great importance. We conclude that the results of these interviews are of importance in the study planning of the intervention SAFE trial ahead, in close collaboration between primary care centres, rehabilitation and community care. For the oldest at high risk of hospitalisation, there seems to be no simple care template.
